# Evaluation of a structured treatment discontinuation in patients with inoperable alveolar echinococcosis on long-term benzimidazole therapy: A retrospective cohort study

**DOI:** 10.1371/journal.pntd.0010146

**Published:** 2022-01-28

**Authors:** Ansgar Deibel, Daniel Stocker, Cordula Meyer zu Schwabedissen, Lars Husmann, Philipp Andreas Kronenberg, Felix Grimm, Peter Deplazes, Cäcilia S. Reiner, Beat Müllhaupt

**Affiliations:** 1 Department of Gastroenterology and Hepatology, University Hospital Zurich, Zurich, Switzerland; 2 Institute of Diagnostic and Interventional Radiology, University Hospital Zurich, Zurich, Switzerland; 3 Department of Nuclear Medicine, University Hospital Zurich, Zurich, Switzerland; 4 Institute of Parasitology, Vetsuisse and Medical Faculty, University of Zurich, Zurich, Switzerland; 5 Graduate School for Cellular and Biomedical Sciences (GCB), University of Bern, Bern, Switzerland; Federation University Australia, AUSTRALIA

## Abstract

**Objectives:**

Alveolar echinococcosis (AE) is an orphan zoonosis of increasing concern in endemic areas, including Europe. It frequently presents in an advanced, inoperable stage, that requires life-long parasitostatic benzimidazole therapy. In some patients, long-term therapy leads to negative anti-Em18 antibody ELISA and PET. It is disputed, whether these patients are truly cured and treatment can be safely discontinued. Our aim was to retrospectively assess long-term outcome of 34 patients with inoperable AE who participated in a previous study to determine feasibility of benzimidazole treatment cessation.

**Methods:**

Retrospective analysis of medical charts was undertaken in all 34 AE patients who participated in our previous study. Of particular interest were AE recurrence or other reasons for re-treatment in patients who stopped benzimidazole therapy and whether baseline clinical and laboratory parameters help identify of patients that might qualifiy for treatment cessation. Additionally, volumetric measurement of AE lesions on contrast-enhanced cross-sectional imaging was performed at baseline and last follow-up in order to quantify treatment response.

**Results:**

12 of 34 patients stopped benzimidazole therapy for a median of 131 months. 11 of these patients showed stable or regressive AE lesions as determined by volumetric measurement. One patient developed progressive lesions with persistently negative anti-Em18 antibody ELISA but slight FDG-uptake in repeated PET imaging. At baseline, patients who met criteria for treatment cessation demonstrated higher lymphocyte count and lower total IgE.

**Conclusion:**

Treatment cessation is feasible in inoperable AE patients, who demonstrate negative anti-Em18 antibody ELISA and PET on follow-up. Close monitoring including sectional imaging is strongly advised.

## Synopsis

Alveolar echinococcosis (AE) is a zoonosis of the liver caused by the larval stage of the fox tapeworm *Echinococcus multilocularis*, which behaves similar to a malignant tumor with mostly infiltrative growth and occasional metastatic spread. Without adequate treatment, AE leads to death in 90% of patients within 10 years. Cure can be achieved through radical surgery, but frequently AE presents in an advanced, inoperable stage, that requires life-long drug therapy. Fortunately, these drugs are very effective. In fact, since their introduction in the mid 1970s, life expectancy of patients with inoperable AE has nearly reached that of the normal population. This leads to the question, whether the parasite has died and treatment can be stopped. Two biomarkers that reflect disease activity are the PET-CT and anti-Em18 antibody. Using these two parameters, we could previously show, that 1/3 of patients might have quiescent disease that allows treatment cessation. In our current study, we provide long-term follow-up of this patient cohort, including lesion size measurements at baseline and last visit. We demonstrate that 11 of 12 patients who stopped therapy show stable disease over a median of 131 months. These results consolidate our approach to a structured treatment interruption in AE.

## Introduction

Alveolar echinococcosis (AE) is an orphan zoonosis caused by the larval stage of the fox tapeworm *Echinococcus multilocularis*. This parasite is predominantly perpetuated in a wildlife-cycle with canines as definitive and small mammals as intermediate hosts. In Switzerland, approximately a 30–70% of the rural or urban fox population are infected, leading to an increase in cases since the new millenium [1,2]. Humans can acquire AE after accidental swallowing of *E*. *multilocularis* eggs [3]. After hatching in the intestine, the oncospheres migrate primarily into the liver, where the larval stage causes a silently progressing hepatic disease [3]. Other organs, including the lungs, kidney, spleen and brain are less often involved [3]. Alveolar echinococcosis behaves similar to a malignant tumor with mostly infiltrative growth and occasional metastatic spread. Without adequate treatment, AE leads to death in 90% of patients within 10 years [4]. Mainstay treatment is surgical resection and medical treatment with benzimidazoles. However, radical surgery is possible only in 20 to 50% of patients due to advanced disease stage at diagnosis [5]. In inoperable AE life-long medical treatment with benzimidazoles is necessary [5].

Benzimidazoles, mainly albendazole and mebendazole, are efficacious in slowing parasite growth and stabilizing the disease [6]. Since their introduction, life expectancy of AE patients has increased significantly [7]. However, long-term benzimidazole therapy is also associated with mild to severe adverse side effects in a considerable portion of patients [8]. In up to one third of patients benzimidazoles may lead to normalization of anti-Em18 antibody enzyme-linked immunosorbent assay (ELISA) and/or perilesionalmetabolic activity in fluorodeoxyglucose positron emission tomography (FDG-PET) imaging [9–12]. The latter quantitatively measures tissue glucose uptake, which is increased in infectious lesions due to the immune cell response [13]. Whether these patients are cured and benzimidazole therapy can be discontinued is still disputed [6,10,14–18]. Currently, there are no other clinical or laboratory parameters that might indicate *in-vivo* parasite viability.

Three studies have addressed the issue of treatment cessation in AE systematically. In their study, Reuter et al. used metabolic activity in PET-imaging after 1 hour as the sole criterium to determine parasite vitality and thereby identify inoperable AE patients that might qualify for treatment cessation [14]. However, they found a high recurrence rate in those patients who were taken off therapy [14]. Caoduro et al. demonstrated feasibility of treatment cessation by use of metabolic activity in PET-imaging after 3 hours and anti-Em2^plus^ antibody ELISA [15]. Both studies had a short follow-up of 2–3 years [14,15]. Our research group used anti-EmII/3-10 (or -Em18, as it was later called) antibody ELISA reactions and metabolic activity in PET-imaging after 1 hour in the selection of patients for treatment interruption and demonstrated stable disease course over 70 months, as determined by persistent Em18 negativity and cross-sectional imaging [11,17].

In the current study, the aims were to determine a) if all patients that stopped treatment showed no disease progression after an extended follow-up, b) how often and why treatment was restarted in these patients, c) if additional patients could stop treatment and d) to identify additional parameters, such as lesion morphology and size, age, sex, treatment type and duration, blood count and chemistry, as well as total immunoglobulin E (IgE), that might help to determine which patients might qualify for treatment cessation.

## Methods

### Ethics statement

Ethical approval for this study was obtained from the local ethics committee (EC) in Zurich (Kantonale Ethikkomission Zürich, EC ZH, BASEC ID: 2020–01169). Patients still followed-up at our clinic provided written consent. For those that had died or were lost to follow-up written consent was waived.

### Study design

All 34 patients who participated in the previous study were included in our current retrospective cohort [17]. The diagnosis of alveolar echinococcosis in all of these patients had been established at least through a suggestive imaging result and two positive serologic tests for echinococcosis, leading to a World Health Organization (WHO) case definition of “probable AE”. In three patients AE had recurred after radical surgery and in another five surgery had achieved only R1 resection. These patients were classified as “confirmed AE” according to WHO case definitions. Patients who had demonstrated negative anti-EmII/3-10 antibody serology and 1h PET imaging after at least 2 years of benzimidazole therapy had qualified for treatment cessation. Patients who had stopped benzimidazole therapy were initially followed-up with repeat anti-EmII/3-10 (-Em18) antibody ELISA every three months and yearly cross-sectional imaging with computed tomography (CT) or magnetic resonance imaging (MRI). After study closure all patients were followed-up with 1–2 yearly anti-EmII/3-10 (-Em18) antibody ELISA and ultrasound. In particular, patients who had stopped benzimidazole therapy additionally received contrast-enhanced cross-sectional imaging (CT or MRI) every 2–3 years. PET imaging was only repeated in case of suspected AE recurrence after treatment cessation or to re-screen patients under benzimidazole therapy for possible treatment cessation.

In case patients were no longer followed up at our clinic, they or their family physicians were contacted to arrange a follow-up visit at our clinic or by phone. Complete follow-up included existing current patient history, imaging report (US, CT or MRI), serology, blood analysis until last contact or study closure date by March 2021. AE-recurrence in patients that stopped treatment was defined as new lesions and/or progression of known lesions on cross sectional imaging with a positive anti-Em18 antibody ELISA or positive PET-CT. Patients who had had to continue benzimidazole treatment during the previous study, were evaluated for possible treatment discontinuation during extended follow-up. Baseline characteristics were acquired from entry into the previous study [17]. The previous study consisted of two groups (A and B) [17]. Group A consisted of 11 inoperable AE patients that had been followed over two years during the initial years of benzimidazole therapy with repeat PET-CT and anti-EmII/3-10 antibody serology, the last series (at two years) decided, whether they qualified for treatment cessation [17]. Group B consisted of 23 patients under long-term benzimdazole therapy, who underwent PET-CT once in order to decide, if they qualified for treatment cessation [17]. In order to compare baseline characteristics from both patients of the A and B group of our previous study, the deciding timepoint of patients from group A was set at two years after study inclusion. This study is reported following the Strengthening the Reporting of Observational Studies in Epidemiology (STROBE) Statement Checklist.

### Data acquisition

Data was acquired through the electronic medical records of the University Hospital Zurich. In case patients were followed-up at a different center, available records were obtained through the local treating physician. In particular, age at diagnosis and screening, PNM classification[19] and stage of the disease, sex, blood count (incl. neutrophil to lymphocyte ratio, NLR) and chemistry (Bilirubin, alanine transaminase [ALT] and alkaline phosphatase [ALP]), total IgE, anti-Em18 antibody ELISA[20], benzimidazole type, prior therapy duration and, in case contrast-enhanced cross-sectional imaging was available, AE lesion size at study inclusion and last follow-up were analyzed and compared between groups. For patients who had been able to stop benzimidazole treatment, time off treatment was calculated and any recommencement of medication and its reason were noted. In case of fatality, cause of death was identified as reported by the death-reporting physician.

### Timing of PET imaging

In our previous study, the metabolic activity of AE lesions was assessed solely 1 hour after flourodeoxyglucose (FDG) administration. Since then, due to the findings of Caoduro et al. [15] protocol was changed to assess activity after 1 and 3 hours. Therefore, PET imaging during extended follow-up was interpreted differently from initial screening. Accordingly, meeting discontinuation criteria also changed. In this study, we report imaging findings after both time points.

### Analysis of cross-sectional imaging

To determine the disease course, volume of the AE lesions was determined as the surrogate marker in addition to Em18 serology. When available at baseline and follow-up, contrast-enhanced cross-sectional imaging was used to measure the volume of the AE lesions. In case of retreatment, last follow-up imaging before re-initation of medical therapy was used. For this, one board certified radiologist (D.S., 6 years of experience in cross sectional imaging), who was blinded to the clinical information, performed volumetric measurements of all hepatic AE lesions on contrast-enhanced MRI, CT or PET-CT studies in the portal venous phase for each patient using dedicated software (Myrian, Intrasense Version 2.6.5). All hepatic lesions were delineated slice by slice using a free-hand region of interest (ROI) tool. Baseline and follow-up exams of the same patient were evaluated side by side in the same session. Cystic and calcified parts of all hepatic AE lesions were included in the volumetric measurements.

### Statistical analysis

Statistical analysis was performed using Graphpad Prism 8 (GraphPad Software, Inc.) Version 8.0.0 (224). To compare non-parametric, unpaired, numerical data the Mann-Whitney U-test was used. For unpaired, categorical data the Fisher’s exact or Chi Square test was applied. A tow-tailed p-value <0.05 was regarded as statistically significant.

## Results

### Baseline characteristics

Age at diagnosis and at screening, sex, stage of AE, prior surgery, lesion size at study inclusion, benzimidazole type and duration of therapy did not differ significantly between the group that initially met criteria for treatment cessation versus the group that did not (Table 1). Interestingly, those patients that met the criteria displayed significantly lower total IgE and significantly higher lymphocyte count (Table 1). Leucocyte, neutrophil and eosinophil counts did not differ significantly (Table 1). The only slight difference of the neutrophil-to-lymphocyte ratio (NLR) did not reach significance (Table 1). Furthermore, patients that met the cessation criteria had slightly but significantly higher baseline ALT levels, although in only 3 of 12 was the ALT above the upper normal limit of normal.

**Table 1:** Baseline characteristics.

		Cessation criteria met at screening (n = 12)	Cessation criteria not met at screening (n = 22)	p-value (univariate)
Age at diagnosis		56y (34-65y)	46y (17-85y)	0.516
Age at screening		64y (48-72y)	60y (24-87y)	0.592
Sex		6 f 6 m	13 f 9 m	0.724
PNM-classification		1 P1N1M0 2 P3N0M0 4 P3N1M0 1 P3N1M1 1 P4N0M0 2 P4N1M0 1 PXN0M0	1 P1N0M0 1 P2N1M0 2 P3N0M0 7 P3N1M0 1 P3N1M1 3 P4N0M0 6 P4N1M0 1 P4N0M1	
Stage		I-II: 0; III-IV: 12	I-II: 1; III-IV: 21	0.999
Recurrence after surgery		2/12	7/22	0.702
Total AE lesion volume		113 cm^3^ (3-519cm^3^, n = 9)	207 cm^3^ (4-1163cm^3^, n = 13)	0.180
Negative Em18 ELISA at screening		12/12	9/22	
Total IgE Titre		36 IU/ml (11–179 IU/ml)	218 IU/ml (6–7440 IU/ml)	0.034*
Blood count at screening				
	Leukocytes	6.54 G/l (4.93–9.97 G/l)	5.70 G/l (3.30–8.35 G/l)	0.125
	Neutrophiles	3.58 G/l (2.96–6.57 G/l)	3.59 G/l (2.08–5.83 G/l)	0.557
	Lymphocytes	1.96 G/l (1.17–3.08 G/l)	1.45 G/l (0.61–2.05 G/l)	0.048*
	Eosinophiles	0.17 G/l (0.09–0.36 G/l)	0.15 G/l (0.02–1.16 G/l)	0.716
	NLR	2.16 (1.09–4.47)	2.63 (1.08–6.79)	0.168
Blood chemistry				
	Bilirubin	10 μmol/l (5–39 μmol/l)	9 μmol/l (5–32 μmol/l)	0.782
	ALT	40 U/l (21–132 U/l)	26 U/l (17–236 U/l)	0.041*
	Alk. Phosphatase	59 U/l (29–418 U/l)	79 (61–329 U/l)	0.138
Benzimidazole therapy		9 ABZ 3 MBZ	11 ABZ 11 MBZ	0.275
Prior treatment duration		59.5 mths (25–240 mths)	98.5 mths (24–316 mths)	0.500

Shown are the baseline characteristics of all 34 patients at screening for our previous study, divided into two groups, depending on whether they did or did not meet treatment cessation criteria.

* p-values <0.05 were regarded as statistically significant.

y = years

m = male

f = female

cm^3^ = cubic centimeters

IU/ml = international units / milliliter

G/l = Giga / liter

ABZ = albendazole

MBZ = mebendazole

mths = months

n.a. = not available.

### Patient follow-up

**Fig 1:**
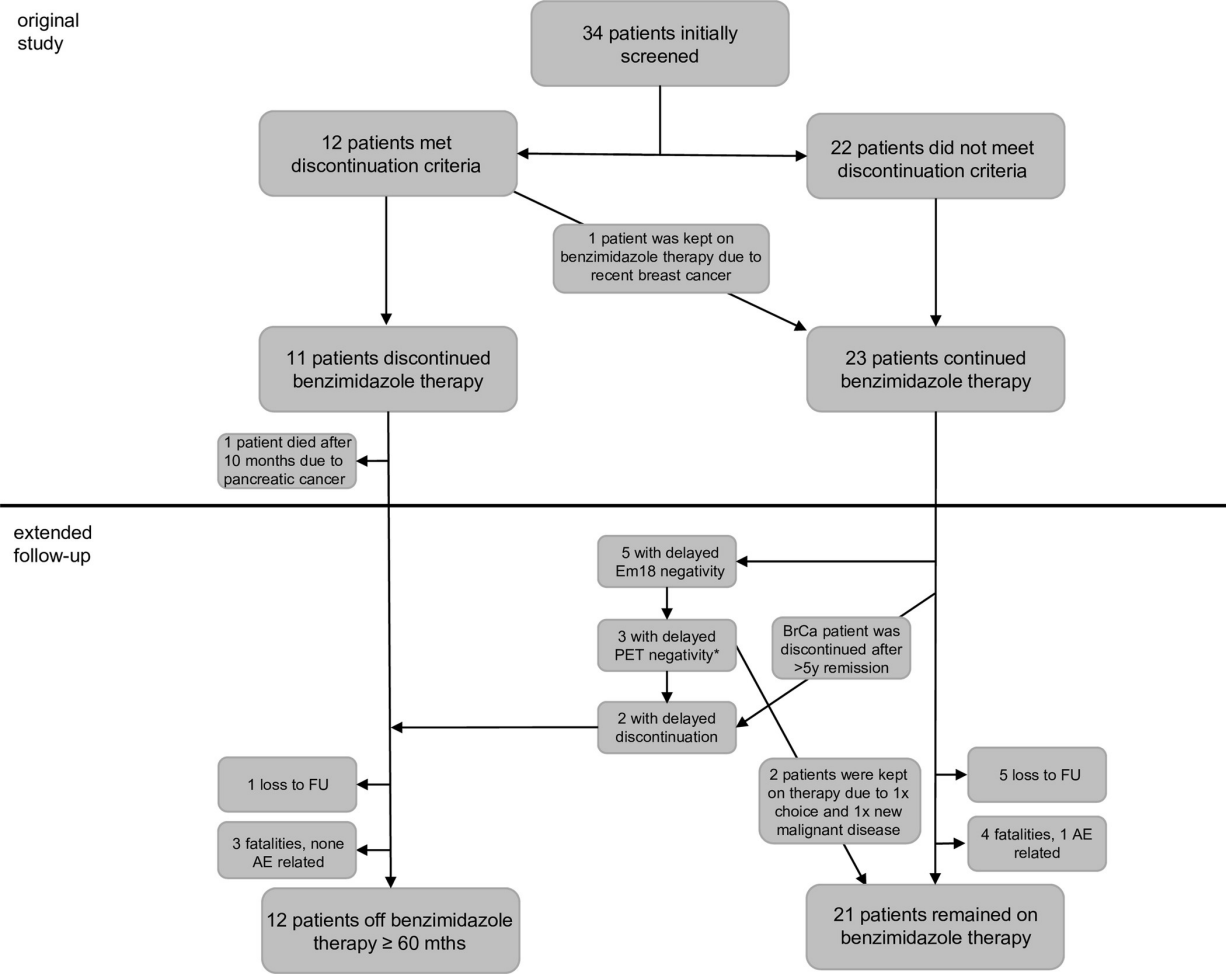
Depicted are the 34 patients who were initially included in our intervention study, to determine outcome after cessation of benzimidazole treatment in serologically and metabolically quiescient disease. 10 patients, who were taken off benzimdazole therapy were followed up at our clinic, 1 moved abroad and was lost to follow-up after 82 months, 3 died of non-AE cause (FU 78, 136 and 172 mths). 5 patients that did not meet cessation criteria showed negative Em18 ELISA on extended follow-up, 3 of them also demonstrated no perilesional FDG-uptake on PET imaging. However, only one was taken off therapy. Reasons to continue therapy were 1x patient choice and 1x incidental finding of metastatic prostate cancer on PET-imaging. In total 12 patients were off therapy for > 60 months (median 131 months).

Medical records of all patients were reviewed. At the end of follow-up four patients in the discontinuation group and five in the treatment continuation group died (Fig 1). Cause of death was related to AE only in one patient who continued benzimidazole therapy (Fig 1, Table 2). One patient, who was off treatment when he died of unknown cause, showed no AE progression at the last follow-up examination five months prior to death. The cause of death in the other 6 patients was pneumonia, heart failure, frailty of old age, spindle cell neoplasm of the liver and unknown (Table 2). Loss to follow-up occurred with only one patient, who discontinued therapy, when he moved abroad after 82 months of uneventful surveillance. Of the group that continued therapy, 5 patients were lost to follow-up after an average of 30 months (Fig 1). Overall, median follow-up time for the treatment discontinuation group was 131 months and 135 months for the treatment continuation group (Table 1). All patients, who had discontinued benzimidazole therapy, displayed persistently negative anti-Em18 antibody ELISA results and lower total IgE at last follow-up (Table 2). Also, there was no difference in the blood levels of ALT, ALP and bilirubin between those patients that stopped and those that continued benzimidazole therapy (Table 2).

Of the group that continued therapy, 5 of the 13 patients, who had not met treatment cessation criteria due to positive anti-Em18 antibody ELISA result, demonstrated delayed anti-Em18 antibody negativity and underwent repeat PET-CT scanning to determine metabolic activity (Fig 1). Two of those patients displayed persistant metabolic activity at 1h and 3h. The other three showed PET negative lesions at 1h and 3h, but only one discontinued therapy (Fig 1). Reason to continue benzimidazole therapy in the other two patients were patient choice and new diagnosis of metastatic prostate cancer (Fig 1). Of the 10 patients, who had not met treatment cessation criteria due to metabolic activity in the screening PET (anti-Em18 antibody ELISA negative), two demonstrated persistent metabolic activity in repeat PET imaging, two opted against repeat PET imaging and one had recurrent anti-Em18 antibody ELISA reaction, the other 5 were lost to follow-up by the time this study was undertaken (Fig 1). Additionally, the patient, who met the criteria for treatment cessation in our previous study but was continued on benzimidazole therapy due to recent diagnosis of breast cancer, was discontinued after cancer-free survival of over 5 years (Fig 1). One patient who had discontinued therapy initially was put on prophylactic treatment before starting chemotherapy for chronic lymphocytic leukemia. All in all, 12 patients were identified who could stop treatment for more than 60 months.

**Table 2:** Extended follow-up.

		Treatment discontinuation (n = 12)	Treatment continuation (n = 21)	p-value (univariate)
Follow-up time, clinical		131 mths (61–172 mths)	136 mths (0–200 mths)	
Follow-up time, imaging		118 mths (61–172 mths; n = 10)	108 mths (55–200 mths, n = 12)	
Negative Em18 ELISA at last follow-up		12/12	16/21	
Blood chemistry at last follow-up				
	Bilirubin	10 μmol/l (5–19 μmol/l)	8 μmol/l (5–62 μmol/l)	0.736
	ALT	26 U/l (12–88 U/l)	19 U/l (9–59 U/l)	0.284
	Alk. Phosphatase	71 U/l (42–203 U/l)	74 (39–115 U/l)	0.766
Total IgE last follow-up		29 IU/ml (9–100 IU/ml)	60 IU/ml (5–3041 IU/ml)	0.032*
Negative repeat PET during follow-up		---	3/6	
AE recurrence		1/12	---	
Re-treatment		2/12 (1 AE recurrence, 1 prophylaxis)	---	
Loss to follow-up		1/12 (FU 82 mths)	5/21	
Deceased patients		3/12	4/21	
Cause of death		• pneumonia (FU 78 mths) • heart failure (FU 136 mths) • unknown (FU 172 mths)	• liver failure due to liver and portal vein thrombosis secondary to AE • spindle cell neoplasm of the liver • frailty of old age • unknown	

Depicted are the characteristics of extended follow-up, dividing patients into two groups, depending on wether they did or did not discontinue therapy. Clinical follow-up time is determined as time between screening and last follow-up. Imaging follow-up time is determined as time between sectional imaging (CT/MRI) at screening and last sectional imaging. Time is shown in months (mths). Negative Em18 ELISA refers to serology testing at last follow-up.

### Image analysis of AE liver lesions

**Fig 2:**
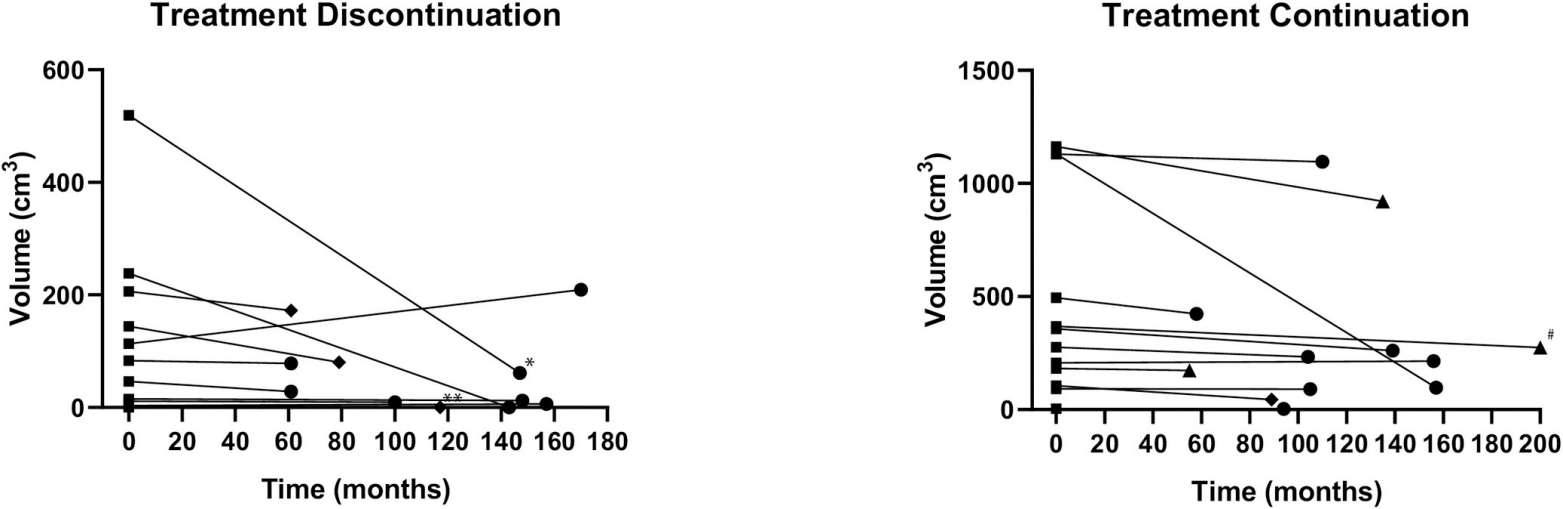
Volumetric measurements. Shown are the AE lesions from 10 patients who discontinued benzimidazole therapy (left: dots and lines), as well as 12 patients who were kept on benzimidazole therapy (right: triangles and dashed lines). Time-points are baseline screening and last sectional imaging follow-up. The median follow-up time was 118 months for the discontinuation group and 111 months for the continuation group. Note that all but one of the patients who discontinued therapy demonstrate a stable or regressive size development. ■ represents the baseline measurements, ● represents follow-up imaging with MRI, ♦ CT and ▲ PET-CT. *this patient had curatively treated cervical cancer stage IIB prior to treatment cessation. **this patient developed chronic lymphocytic leukemia, shown is the lesion volume prior to reinstated prophylactic benzimidazole therapy. ^#^this patient had HIV, adequately controlled with anti-retroviral therapy and normal CD4 lymphocyte count.

**Fig 3:**
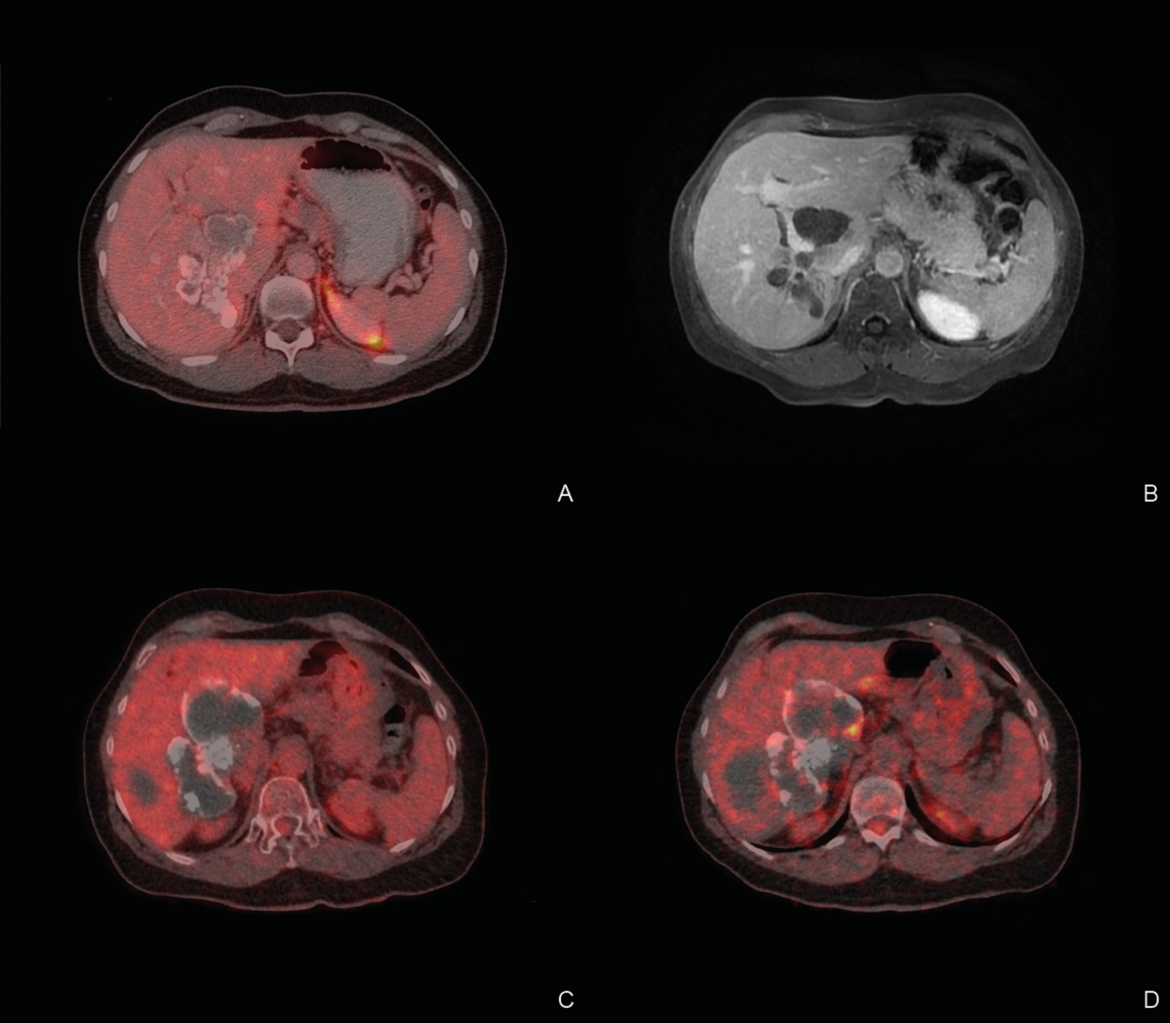
Imaging follow-up of the only patient that demonstrated a progressive disease course after cessation of benzimidazole treatment. A: PET-CT at baseline. B: MRI after 4 years off therapy. C: PET-CT after 14 years off-therapy. Imaging 1h after FDG injection. D: Same PET-CT as in C but imaging 3h after FDG injection. Note the discret hilar FDG uptake.

Of the 12 patients, with discontinued treatment for more than 60 months, 10 demonstrated AE lesions that could be measured. One of the other two patients demonstrated only very small calcifications in the liver, which were not suitable for volumetric measurement. The other patient was followed-up at a different center with ultrasound imaging only. Median follow-up time for cross-sectional imaging was 118 months, ranging from 61–172 months (Table 2). Over this time period, 9 of the 10 patients with measurable AE lesions demonstrated either a stable or decreasing lesion size (Fig 2). In contrast, in one patient the lesions almost doubled in size, mainly through growth of a cystic part in liver segment IV (Figs 2 and 3). Repeat Em18 ELISA of this patient’s serum, however, was consistently negative and repeat PET-CT demonstrated no metabolic activity after 1h and only discrete hilar activity at 3h (Fig 3).

Of the 21 patients that continued benzimidazole therapy throughout the entire study period, 12 had received contrast-enhanced cross-sectional imaging at baseline and at some timepoint throughout extended follow-up, which was available for volumetric measurement. Median follow-up for cross-sectional imaging was 108 months, ranging from 55–200 months (Table 2). All patients demonstrated either a stable or regressive course of the AE lesion size (Fig 2).

## Discussion

The primary aim of this study was to evaluate the currently still disputed structured approach to treatment cessation in patients with inoperable AE by retrospective analysis of the extended follow-up of all patients included in our previous study [17]. To the best of our knowledge, this is the first study to analyse lesion volumetry in patients who discontinued benzimidazole therapy and reports the longest follow-up after treatment cessation.

Our analysis shows, that almost all patients who met the criteria for treatment cessation showed a favorable outcome after a median of 10.9 years off benzimidazole therapy. This is underlined by our finding, that 11 of the 12 patients showed a stable or decreasing AE lesion volume and persistently negative anti-Em18 antibody ELISA results. Only one patient demonstrated an increase of the AE lesion size. It is important to note, that in between treatment cessation and last follow-up for the current study, this patient chose to have follow-up ultrasound at a center without a specialist in AE for 9 years. Repeated anti-Em18 antibody ELISA was persistently negative but repeated PET imaging showed very slight FDG-uptake (standardized uptake value [SUV] of 5.9) only after 3h. Accordingly, the patient was restarted on benzimidazole therapy.

In conclusion, the selection of patients with inoperable AE for treatment cessation after at least two years of benzimidazole therapy and negative anti-Em18 antibody ELISA and PET on follow-up is feasible. Depending on the local availability of serological markers and AE center experience, anti-Em2^plus^ antibody ELISA may be used alternatively, as it has been shown to also reflect disease activity [15,21]. When discontinuing benzimidazol therapy, two important points have to be kept in mind, as highlighted by the one patient with late progression of the disease in this study. First, both biomarkers do not reflect the parasite viability but rather the immune response to it. Therefore, we suggest to avoid the term “parasitocidal efficiacy” of benzimidazol therapy in patients that meet cessation criteria, as it gives false certainty that recurrence of disease is no longer possible. Rather, it should be viewed that activity of the disease has been reduced to a minimum, i.e. quiescent disease, that allows treatment cessation under continued surveillance. Second, anti-Em18 antibody ELISA serology alone is not sufficient to monitor patients, as it can stay negative in some patients despite disease recurrence as shown here and in our previous study [11]. On the other hand, adding repeat PET imaging is invasive, costly and the ideal timing and interpretation of FDG-uptake measurement debated [15,16]. We therefore recommend to follow these patients up at an AE specialist center with repeat cross-sectional imaging to accurately assess lesion growth.

It is also important to note that in our previous prospective study, PET imaging reported measurement of FDG-uptake only after 1 hour. As shown by Caoduro et al., delayed PET imaging after 3 hours is more sensitive for the detection of perilesional metabolic activity in AE than after conventional 1 hour [15]. However, to date it is unclear, whether and to what extend perilesional FDG-uptake correlates with disease activity and lesion growth. Tighening of selection criteria by extending PET-imaging to 3 hours could unnecessarily prolong benzimidazole therapy [16]. Further studies are therefore needed in order to accurately determine the value of delayed PET imaging in the selection of patients for treatment cessation.

The secondary aim of this study was to find further clinical and/or laboratory parameters that might help to distinquish AE patients that might qualify for treatment cessation. At baseline, only total IgE and lymphocyte count differed significantly between those who met criteria and those who did not. The role of periphal blood lymphopenia in AE has not been evaluated systematically. In cancer, lymphopenia has been associated with poorer prognosis and survival [22,23]. As indicated by our findings, similar implications could be valid in AE, although, it might also reflect a potential genetic component of the disease course. The relevance of low total IgE in AE is equally unclear. In cystic echinococcosis (CE), IgE and CE-specific IgE have been shown to reflect disease activity [24,25]. According to our findings, high total IgE could reflect on-going disease activity. However, further prospective studies with a larger patient cohort are needed to determine the usefulness of the two biomarkers as predictors in the disease course of AE.

Naturally, there are some limitations to this study. In particular, the retrospective nature of data analysis, the lack of a predetermined surveillance protocol, some patients being lost to follow-up and some lacking contrast-enhanced sectional imaging that could be used for volumetric measurements. However, data on the most important group, being those that discontinued benzimidazole therapy, was mostly complete.

## Conclusion

Cessation of benzimidazole therapy in inoperable but metabolically (PET) and serologically (anti-Em18 antibody ELISA) quiescient alveolar echinococcosis is possible with a favourable outcome, but careful follow-up with regular cross-sectional imaging in a specialist center for AE treatment is mandatory.
